# LncRNA PVT1 in human cancers: genomic complexity, isoforms, functional elements, mechanism of action, subcellular localization and possible role as a therapeutic target

**DOI:** 10.1186/s12943-026-02576-y

**Published:** 2026-01-21

**Authors:** Chen Li, Francisco Alejandro Lagunas-Rangel, Lutao Du, Chengxi Sun, Helgi B. Schiöth

**Affiliations:** 1 Department of Clinical Laboratory, The Second Qilu Hospital of Shandong University, 247 Beiyuan Street, Jinan, Shandong 250033 China; 2 Department of Surgical Sciences, Functional Pharmacology and Neuroscience, Uppsala University, Uppsala, Sweden; 3 Laboratory of Pharmaceutical Pharmacology, Latvian Institute of Organic Synthesis, Riga, Latvia; 4 Department of Clinical Laboratory, Qilu Hospital of Shandong University, Jinan, China; 5 Department of Clinical Laboratory, Qilu Hospital of Shandong University, No. 107 Wenhua West Road, Lixia District, Jinan, Shandong 250012 China; 6 Department of Surgical Sciences, Functional Pharmacology and Neuroscience Biomedicinskt Centrum, Uppsala University, Husargatan 3, Uppsala, 753124 Sweden

**Keywords:** MiRNA sponging, PVT1, Cancer mechanisms, Antagomirs, CRISPR/Cas9 therapeutics

## Abstract

PVT1 (Plasmacytoma Variant Translocation 1), a long non-coding RNA (lncRNA), is a critical player in diverse biological processes, particularly cancer biology. This review comprehensively examines PVT1’s multifaceted roles and molecular mechanisms. We delve into its predominant oncogenic functions while highlighting notable exceptions, offering new perspectives on its biological diversity. A key focus is the subcellular localizations of PVT1 across various cancers – an aspect underexplored in previous reviews despite its significant impact on function and regulatory mechanisms. Detailed mechanistic insights include PVT1’s linear and circular isoforms acting as miRNA sponges, its encoding of miRNAs, the influence of 8q24 rearrangements, and its regulation of protein stability/activity, and transcription. Furthermore, we examine PVT1’s functional elements, illuminating unexplored structural and regulatory features. The clinical implications of PVT1 as a biomarker and therapeutic target in oncology are also discussed. By synthesizing existing knowledge and introducing previously less-explored facets, this review aims to provide a comprehensive and up-to-date resource on PVT1’s role in health and disease.

**Supplementary Information:**

The online version contains supplementary material available at 10.1186/s12943-026-02576-y.

## Introduction

Long non-coding RNAs (lncRNAs) are a diverse class of RNA molecules longer than 200 nucleotides that, although non-protein-coding, are critical regulators of gene expression and various cellular processes [1, 2]. Given their broad regulatory potential, lncRNAs are implicated in numerous diseases, including cancers, cardiovascular diseases, and neurodegenerative disorders [3–19]. Recent advances in transcriptomics have unveiled thousands of lncRNAs, but only a fraction has been functionally characterized. Their specificity to certain tissues and disease states makes them promising targets for biomarker discovery and therapeutic intervention. Studying lncRNAs offers valuable insights into the complexity of gene regulation, potentially leading to new avenues in personalized medicine.

The Plasmacytoma Variant Translocation 1 (PVT1) gene locus gained significance in the early 1980 s with the discovery of variant (6;15) translocations in murine plasmacytomas. These translocations entail the fusion of the myc oncogene-bearing segment of chromosome 15 with the immunoglobulin kappa segment of chromosome 6. Notably, the breakpoints of these translocations do not occur proximal to c-myc, highlighting the crucial role of the pvt-1 gene locus as the primary site for such translocations [20]. The pvt-1 gene product likely contributes to the activation of c-myc expression triggered by these translocations [20]. Moreover, pvt-1 serves as a major site for retroviral insertions in murine T lymphomas, potentially promoting T cell neoplasia by influencing c-myc expression over long distances [21]. The mouse pvt-1 region shares homology with a corresponding region in rats called Mis-1, which also acts as a frequent site for retroviral integration in rat T lymphomas [22]. This conservation extends to humans, where the 2;8 variant translocation observed in Burkitt’s lymphomas closely resembles the t (6;15) found in murine plasmacytomas. Both involve homologous major breakpoint regions on chromosome 8 in humans and chromosome 15 in mice, specifically within the PVT1 locus [23]. This conservation across species underscores the functional importance of the PVT1 locus.

In humans, the PVT1 gene spans over 300 kb and is situated 55 kb away from the MYC gene locus on chromosome 8q24.21, a region well-known for its association with cancer development. Gene rearrangement results in aberrant expression of PVT1 and MYC, with a closely linked genomic phenomenon being gene amplification. The amplification of this genomic region, often triggered by genomic instability in cancer cells, is a common observation in various human cancers [24–31]. In more than 98% of cases where the copy number of MYC increases, there’s a simultaneous increase in the copy number of PVT1, which is correlated with a poorer prognosis [32]. In cancer cells, the expression levels of PVT1 and MYC are typically correlated. MYC can activate the transcription of PVT1 [33, 34], while PVT1 can stabilize the MYC protein [32]. The simultaneous amplification and upregulation of these two genes often lead to synergistic pro-oncogenic effects, such as increased cell proliferation, suppression of apoptosis, promotion of angiogenesis, and heightened metastatic potential.

Following the discovery of PVT1’s association with cancer, there has been an explosion of cancer-related research on this molecule. PVT1 has been implicated in nearly all forms of human cancers; nevertheless, its precise mechanism of action remains predominantly enigmatic across various cancer types. The intricate nature of the PVT1 gene locus poses a significant challenge in elucidating the precise functional elements and molecular mechanisms driving its role in cancer. This complexity stems from over 35 linear PVT1 isoforms generated through alternative splicing, alongside circular RNA (circRNA) and a cluster of miRNAs.

Currently, there are several reviews focused on PVT1, as well as on some of its products such as circPVT1 or its derived miRNAs. However, none of these studies pay significant attention to the subcellular localization of these RNAs, despite their potential impact on their function and regulatory mechanisms. In this review, we address the complexity of the PVT1 gene, its ability to encode dozens of isoforms, and its varied roles and mechanisms of action across different cancer types. We investigate how the mechanism of action of PVT1 lncRNA correlates with its subcellular localization and present the first comprehensive overview of PVT1’s localization patterns in diverse cell lines. Additionally, we summarize its known functional elements, providing a critical foundation for developing PVT1-targeted clinical interventions. Moreover, we analyze PVT1’s diverse mechanisms of action across various cancers, highlighting its multifaceted roles in tumor biology. Finally, this review emphasizes PVT1’s potential as both a clinical biomarker and a therapeutic target, underscoring its significance for advancing drug discovery and translational oncology research.

## Approach for data collection and search

A comprehensive literature review was conducted using the NCBI-National Library of Medicine-PubMed database (https://pubmed.ncbi.nlm.nih.gov/), encompassing articles published up to March 2025. The primary search term, PVT1 (Plasmacytoma Variant Translocation 1), was employed to identify relevant studies. Articles were screened and selected based on predefined inclusion criteria, with a focus on the cancer-related roles, subcellular localization, and molecular mechanisms of PVT1. This rigorous approach laid a robust foundation for an in-depth exploration of PVT1’s biological functions and mechanistic pathways.

## PVT1’s dominance in cancer promotion: exploring remarkable exceptions

**Fig. 1:**
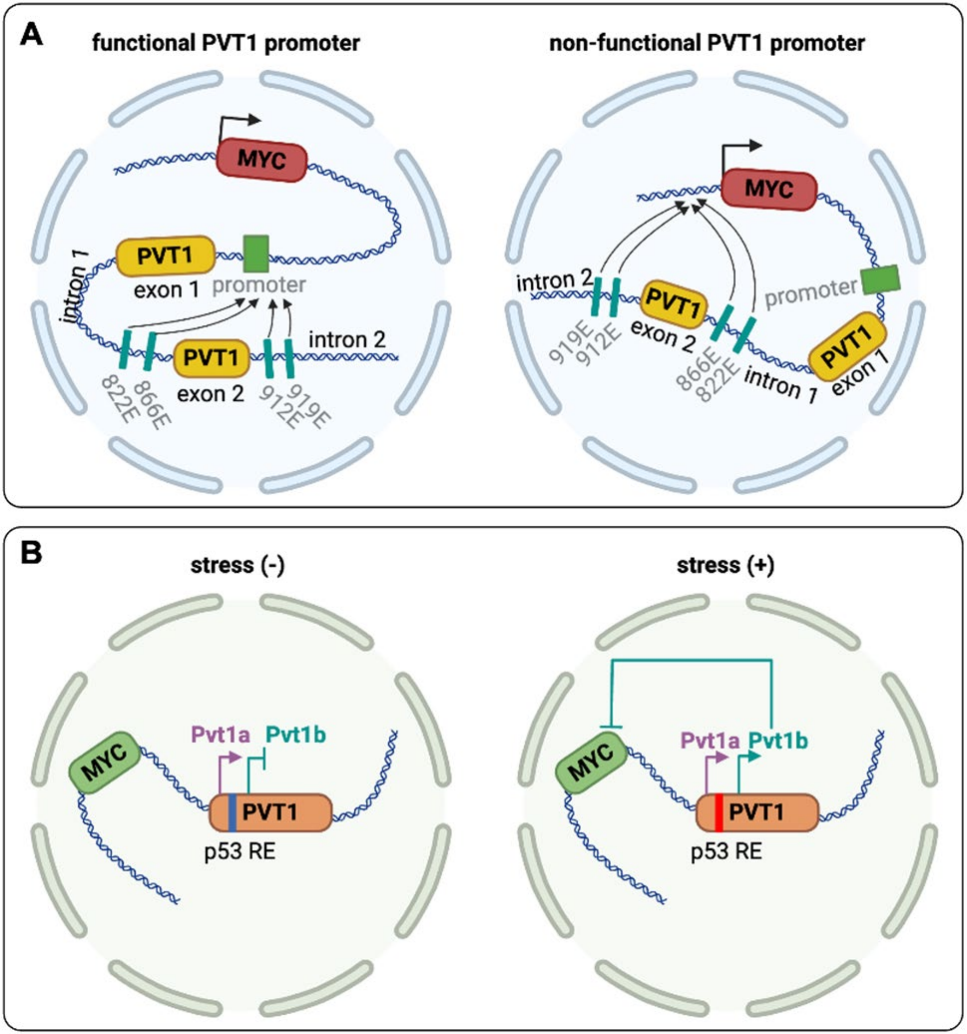
The tumor suppressive role of PVT1. **A** Four intragenic enhancers within the PVT1 locus, named 822E, 866E, 912E, and 919E, normally preferentially contact the PVT1 promoter over the MYC locus (functional PVT1 promoter; left). CRISPR interference of PVT1 decreased the contacts of these four enhancers with the PVT1 promoter but increased contacts with the MYC promoter and a MYC 3′-enhancer (non-functional PVT1 promoter; right). **B** In unstressed tumor cells, the transcription of Pvt1a takes precedence over Pvt1b (left). In tumor cells exposed to genotoxic and oncogenic stressors, the transcription of Pvt1b is triggered by p53. This activation of Pvt1b suppresses Myc transcription *in cis* during the cellular stress response (right)

PVT1 has been the subject of extensive research in the context of cancer, with numerous studies demonstrating its pivotal role in driving cancer progression. PVT1 modulates key cellular mechanisms such as proliferation, apoptosis, migration, and invasion across various human cancers, including but not limited to, gastric cancer, colorectal cancer, hepatocellular carcinoma, pancreatic cancer, lung cancer, bladder cancer, ovarian cancer, endometrial carcinoma, and glioma. Nevertheless, the oncogenic partnership between MYC and PVT1 has become increasingly intricate due to the suppressive actions of the PVT1 promoter [35] and the p53-activated-PVT1b isoform [36] on MYC transcription. Cho et al. [35] identified a tumor-suppressive effect linked to the promoter region of the PVT1 gene in breast cancer. Their research revealed that CRISPR interference targeting the PVT1 promoter enhances breast cancer cell competition and growth in vivo. The promoters of PVT1 and the MYC oncogene vie for interaction with four intragenic enhancers within the PVT1 locus. When the PVT1 promoter is silenced, it paves the way for heightened interaction between MYC and the enhancers within PVT1, consequently triggering increased transcription of MYC. Notably, the tumor-suppressive role of the PVT1 promoter operates independently of the PVT1 lncRNA (Fig. 1A). On the other hand, Olivero et al. [36] identified a conserved isoform of Pvt1, Pvt1b, which plays a pivotal role in inhibiting cellular proliferation and curtailing tumor growth in lung cancer. Pvt1b can be promptly triggered by p53 in reaction to both genotoxic and oncogenic stressors, consequently efficiently inhibiting Myc transcription. The activation of Pvt1b serves to restrain the proliferation of lung cancer cells and restrict tumor expansion during the initial stages of lung cancer progression (Fig. 1B). The PVT1 gene locus has remained a focal point of investigation due to its involvement in a multitude of biological processes, notably tumor development. The aforementioned study reinforces the intricate and diverse nature of this gene locus in its function and interaction with other genes.

## Subcellular localizations of PVT1

LncRNAs exert profound influence on gene expression across multiple levels, including the epigenetic, transcriptional, and post-transcriptional levels, by engaging with proteins, DNA, and RNA molecules. The subcellular localization of lncRNAs is a fundamental determinant of their function, as it dictates their interaction partners and spatiotemporal dynamics. Nuclear lncRNAs predominantly govern epigenetic modifications and transcriptional activities, while cytoplasmic lncRNAs are key regulators of post-transcriptional processes such as mRNA stability and translation. Consequently, understanding the precise subcellular compartmentalization of lncRNAs is crucial for deciphering their biological roles.

Experimental approaches for determining the subcellular localization of lncRNAs commonly involve cell fractionation, in situ hybridization (ISH), and fluorescence in situ hybridization (FISH). Applying these methods, studies have revealed that the oncogenic lncRNA PVT1 exhibits distinct and often tissue-specific subcellular distributions (Table 1). For example, linear PVT1 transcripts mainly localize within the nucleus in cancers such as colorectal cancer [37, 38], glioblastoma multiforme [39], cholangiocarcinoma [40], gastric cancer [41], gallbladder cancer [42], lung adenocarcinoma [43], nasopharyngeal carcinoma [44] and in breast cancer [45]. In contrast, PVT1 is found primarily in the cytoplasm in head and neck squamous cell carcinoma [46]. Notably, conflicting reports exist regarding PVT1 localization in cutaneous squamous cell carcinoma [34, 47], which may reflect inherent sensitivity differences among the various localization assays employed.

**Table 1:** Subcellular localizations of PVT1

Cancer/sample	Subcellular localization	Function	Method	References
colorectal cancer/HCT116 and LoVo cells	circPVT1: mainly cytoplasmic most of linear PVT1: mainly nuclear		cell fractionation	[37]
colorectal cancer/HCT116	linear PVT1: mainly nuclear	acts as oncogenic enhancer of MYC	cell fractionation	[38]
glioblastoma multiforme/U87 and U251 cells	linear PVT1: mainly nuclear	interacts with DHX9 and modulates expression of type I interferon-stimulated genes and CX3CL1	cell fractionation FISH	[39]
cholangiocarcinoma/HuCCT1 and RBE cells	linear PVT1: mainly nuclear	binds to PRC2 and adjusts the histone methylation of the promoter of ANGPTL4	cell fractionation FISH	[40]
gastric cancer/SGC-7901 and BGC-823 cells	linear PVT1: mainly nuclear	epigenetically regulates the expression of p15 and p16 by modulating EZH2 activity	cell fractionation	[41]
gallbladder cancer/GBC-SD and SGC-996 cells	linear PVT1: mainly nuclear	recruitfs DNMT1 via EZH2 to the miR-18b-5p DNA promoter and suppresses the transcription of miR-18b-5p	cell fractionation	[42]
lung adenocarcinoma/H1299 and HCC827 cells	linear PVT1: mainly nuclear	recruits EIF4A3 to enhance circLMNB2 expression	cell fractionation	[43]
nasopharyngeal carcinoma/HNE-1 and CNE-1 cells	linear PVT1: mainly nuclear	acts as a scaffold for the chromatin modification factor KAT2A, mediating H3K9 acetylation and recruiting TIF1β to activate NF90 transcription	cell fractionation	[44]
cutaneous squamous cell carcinoma/tissue sections	linear PVT1: mainly nuclear		RNAscope	[34]
breast cancer/MCF-7	linear PVT1: mainly nuclear	acts as a bridge between ERα and PRC2 and inhibits the expression of pro-apoptotic genes and tumor suppressor genes	cell fractionation	[45]
head and neck squamous cell carcinoma/HN6 cells	linear PVT1: mainly cytoplasmic	miRNA sponge for miR-375	cell fractionation	[46]
cutaneous squamous cell carcinoma/A431 and COLO16 cells	linear PVT1: mainly cytoplasmic	binds and regulates 4EBP1 protein expression	cell fractionation FISH	[47]
breast cancer/MCF-10 A cells	circPVT1: mainly cytoplasmic	inhibits the function of miR-33a-5p through sponge adsorption, releases the activity of c-MYC, thereby activating GLS1 transcription	cell fractionation	[48]

Critically, lncRNAs'f subcellular localization is dynamically regulated through coordinated molecular mechanisms rather than passive diffusion [49, 50]. Specific cis-elements in lncRNAs, such as exonic repeats (e.g., RIDLs including L1PA16, L2b, MIRb, and MIRc) [51], C-rich motifs (e.g., SIRLOIN) [52], U1-binding motifs [53], and R-loop structures formed by antisense lncRNAs [54, 55], play crucial roles in promoting nuclear localization. These cis-elements can interact with various trans-factors, including hnRNPs (e.g., hnRNPU interacts with RRD in Firre [56, 57], hnRNPK binds to C-rich motifs in SIRLOIN [52]), RNA helicases (e.g., DHX15, DDX42 [58]), and splicing factors (e.g., U1-snRNP associated with U1-binding motifs [59]). For instance, the SIRLOIN element in lncRNA PVT1 binds HNRNPK, driving nuclear accumulation [52]. Conversely, lncRNAs lacking nuclear retention elements are exported to the cytoplasm. Additionally, GC-rich RIDLs are associated with cytoplasmic enrichment of lncRNAs [51]. Circular RNAs (circRNAs) that are longer than 800 nucleotides can be efficiently exported to the cytoplasm through UAP56, whereas shorter circRNAs (less than 800 nucleotides) are exported through URH49 [60].

Importantly, the compartment-specific localization of PVT1 dictates its divergent functional roles in cancer pathogenesis. Nuclear PVT1 orchestrates chromatin-centric oncogenesis through context-dependent interactions: In colorectal cancer, it amplifies MYC oncogenicity through acting as oncogenic enhancer of MYC [38]; in glioblastoma, it complexes with DHX9 to dysregulate type I interferon-stimulated genes and CX3CL1 [39]; in cholangiocarcinoma, it recruits PRC2 to modulate ANGPTL4 promoter histone methylation [40]; in gastric cancer, it epigenetically silences p15 and p16 by modulating EZH2 activity [41]; in gallbladder cancer, it recruits DNMT1 through EZH2 to suppress miR-18b-5p transcription [42]; in lung adenocarcinoma, it recruits EIF4A3 to enhance circLMNB2 biogenesis [43]; in nasopharyngeal carcinoma, it acts as a scaffold for the chromatin modification factor KAT2A, mediating H3K9 acetylation and recruiting TIF1β to activate NF90 transcription [44]; and in breast cancer, it acts as a bridge between ERα and PRC2 and inhibits the expression of pro-apoptotic genes and tumor suppressor genes [45]. Conversely, cytoplasmic PVT1 predominantly executes non-chromatin oncogenic functions: It serves as a master ceRNA regulator—sponging miR-375 to derepress YAP1 expression in head and neck squamous cell carcinoma [46]; sponging miR-33a-5p to release the activity of c-MYC and activate GLS1 transcription [48]; while in cutaneous squamous cell carcinoma (cSCC) it post-transcriptionally regulates 4EBP1 protein expression to promote tumorigenesis [47].

## Molecular mechanisms of PVT1

### Linear and circPVT1 act as MiRNA sponges

**Fig. 2:**
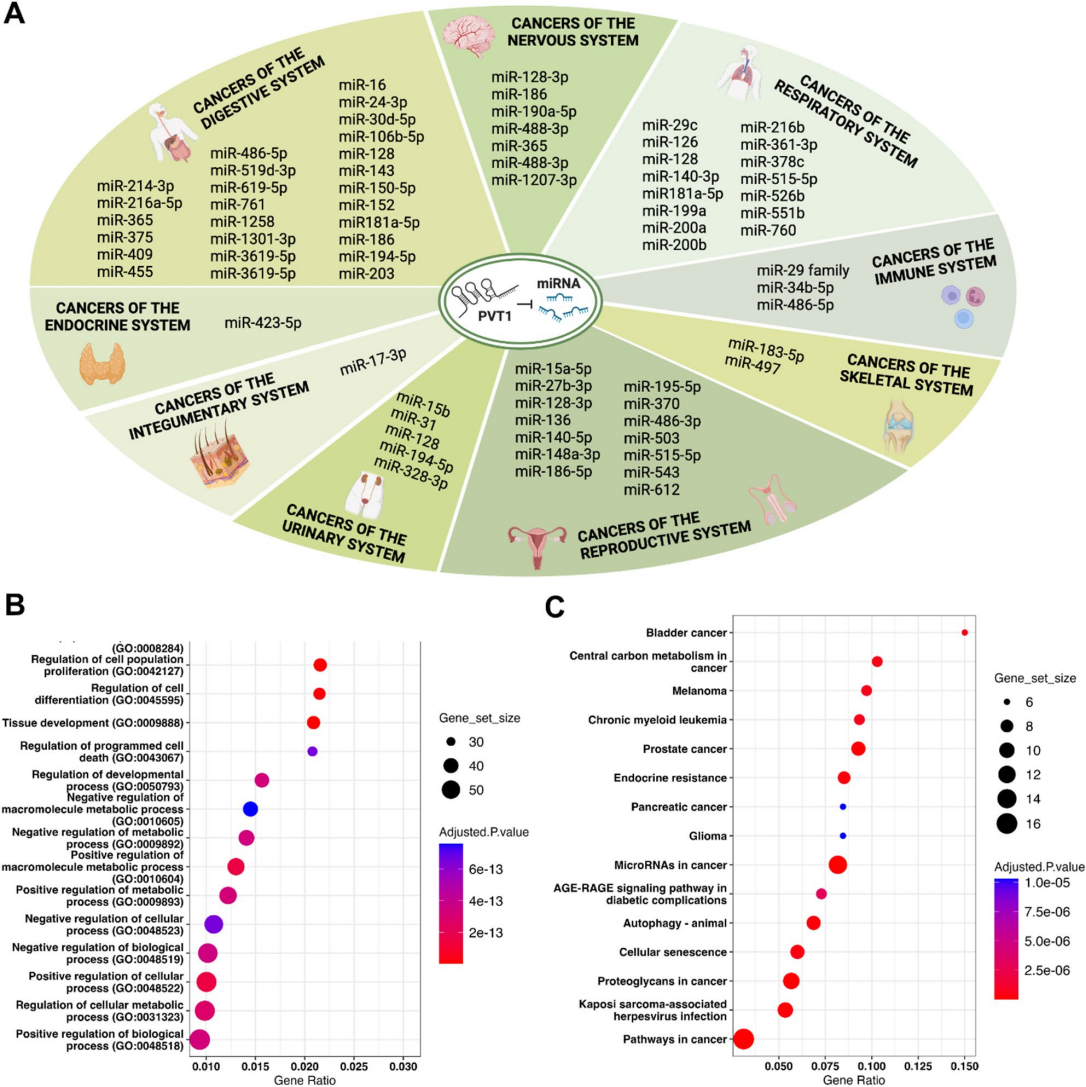
Linear PVT1 acts as miRNA sponges. **A** PVT1 functions as miRNA sponges, sequestering miRNAs to regulate downstream target mRNAs. The diagram categorizes the miRNAs bound by PVT1 according to various cancer systems, including cancer of the digestive, nervous, respiratory, immune, skeletal, reproductive, urinary, integumentary, and endocrine systems. More detailed information on cancer types, target genes, and the regulation of cancer cell phenotypes is provided In Supplementary Table 1. **B**,**C** The top fifteen pathways significantly enriched among the target genes of PVT1-binding miRNAs in the GO Biological Processes (**B**) and KEGG database (**C**) (FDR < 0.01). The color of the nodes indicates the adjusted P-value, and the size of the nodes reflects the number of overlapping genes in the gene sets

PVT1 has been discovered to serve as a sponge for various miRNAs. Through its binding affinity with miRNAs, lncRNA PVT1 effectively modulates the stability and functionality of these miRNAs, thereby exerting influence over the expression of their target genes. The sequestration of miRNAs by lncRNA PVT1 is a common phenomenon in various cancer types across different systems (Fig. 2A). LncRNA PVT1 sequesters different miRNAs to regulate distinct target mRNAs (Supplementary Table 1) [46, 61–141]. Enrichment analysis of these target mRNAs reveals their involvement in biological processes such as cell proliferation, differentiation, programmed cell death, and cellular metabolism (Fig. 2B). Additionally, KEGG enrichment analysis indicates that these target mRNAs are cancer-related and associated with cellular autophagy and senescence (Fig. 2C).

Similarly, circRNA PVT1, derived from the same genomic locus as lncRNA PVT1, also acts as a sponge for miRNAs. CircRNAs are distinguished by their covalently closed loop structure, rendering them resistant to degradation by RNA exonucleases and thus more stable than linear RNAs. In this capacity, circRNA PVT1 effectively sequesters miRNAs, thereby regulating their activity and contributing to the intricate regulatory network governing cellular processes such as proliferation, apoptosis, and metastasis. Consequently, circRNA PVT1 plays a pivotal role in the initiation and progression of cancer (Table 2).

**Table 2:** circPVT1 acts as MiRNA sponges

Cancer	Interacting miRNA	Target gene	Function	References
laryngeal cancer	miR-21-5p	CBX4	promote proliferation, migration, and invasion; inhibit apoptosis	[142]
osteosarcoma	miR-24-3p	KLF8	promote proliferation and cisplatin resistance	[143]
breast cancer	miR-29a-3p	AGR2	promote cell growth, invasion, migration; inhibit apoptosis	[144]
lung squamous cell carcinoma	miR-30d/e	CCNF	promote cell proliferation and xenograft tumor growth in vivo	[145]
oral squamous cell carcinoma	miR-106a-5p	HK2	promote cell growth, metastasis and glycolysis	[146]
gastric cancer	miR-124-3p	ZEB1	contribute to paclitaxel resistance in vitro and in vivo	[147]
oral squamous cell carcinoma	miR-125b	STAT3	promote proliferation	[148]
osteosarcoma	miR-137	TRIAP1	contributes to doxorubicin resistance; promote tumor growth in vivo	[149]
oral squamous cell carcinoma	miR-143-3p	SLC7A11	promote proliferation, migration, and invasion; inhibit apoptosis	[150]
clear cell renal cell carcinoma	miR-145‐5p	TBX15	promote proliferation and invasion; promote growth and metastasis in vivo	[151]
lung adenocarcinoma	miR-145-5p	ABCC1	contribute to cisplatin and pemetrexed resistance	[152]
ovarian cancer	miR-149-5p	FOXM1	promote proliferation and invasion	[153]
gastric cancer	miR-152-3p	HDGF	contribute to cisplatin resistance	[154]
ER-positive breast tumorigenesis	miR-181a‐2‐3p	ESR1	promote cell growth; circPVT1 directly interacts with MAVS protein to disrupt the RIGI–MAVS complex formation, inhibiting type I interferon (IFN) signaling pathway and anti-tumor immunity	[155]
gastric cancer	miR-195-5p	ETS1	promote proliferation and invasion; inhibit apoptosis	[156]
papillary thyroid carcinoma	miR-195	VEGFA	activate the Wnt/β-catenin signaling pathway; promote growth, migration, and invasion	[157]
hepatocellular carcinoma	miR-203	HOXD3	promote proliferation and migration; promote tumor growth in vivo	[158]
hepatocellular carcinoma	miR-377	TRIM23	promote proliferation and glycolysis; inhibit apoptosis; promote tumor growth in vivo	[159]
esophageal squamous cell carcinoma	miR-423–5p	BTRC	switch on NF-κB signaling pathway via diminishing IKBA expression; promote proliferation and cell cycle	[160]
gastric cancer	miR-423-5p	SMAD3	promote cell growth, invasion, migration and EMT	[161]
osteosarcoma	miR-423‐5p	Wnt5a and Ror2	promote glycolysis, proliferation, migration, and invasion	[162]
lung adenocarcinoma	miR‑429	FOXK1	contribute to cisplatin resistance; promote cell viability, proliferation, invasion and migration	[163]
thyroid cancer	miR-455-5p	CXCL12	promote proliferation, migration, and invasion; promote tumor growth and lung metastasis in vivo	[164]
non-small cell lung cancer	miR-497	Bcl-2	promote proliferation; inhibit apoptosis; promote tumor growth in vivo	[165]
osteosarcoma	miR-526b	FOXC2	promote metastasis	[166]
hepatocellular carcinoma	miR-3666	SIRT7	promote cell growth	[167]

### Encoding MiRNAs

In addition to acting as miRNA sponges, the PVT1 gene locus encodes several miRNAs, including miR-1204, miR-1205, miR-1206, miR-1207-3p, miR-1207-5p, and miR-1208 [168]. The miRNAs encoded by the PVT1 gene locus demonstrate tissue-specific expression patterns, indicating that their expression levels and functions vary depending on the tissue type where they are active. For example, miR-1204, miR-1205, miR-1207-5p, and miR-1208 are expressed in malignant pleural mesothelioma cell lines, while miR-1206 and miR-1207-3p are not [30]. In prostate cancer cell lines, miR-1204, miR-1205, miR-1206, miR-1207-3p, and miR-1208 are expressed, whereas miR-1207-5p is not [169]. Among gastric tumor tissues, adjacent non-neoplastic gastric tissues from patients with gastric cancer, and non-neoplastic gastric tissues from individuals without gastric cancer, miR-1205, miR-1207-3p, miR-1207-5p, and miR-1208 are all expressed, while miR-1204 and miR-1206 are not [170] (Table 3).

**Table 3:** Expression of MiRNAs encoded by the PVT1 gene locus in different cell lines

	miR-1204	miR-1205	miR-1206	miR-1207-5p	miR-1207-3p	miR-1208
**malignant pleural mesothelioma** [30]						
H28	**+**	**+**	**-**	**+**	**-**	**+**
H2452	**+**	**+**	**-**	**+**	**-**	**+**
HP10	**+**	**+**	**-**	**+**	**-**	**+**
HP7	**+**	**+**	**-**	**+**	**-**	**+**
HCT-4012	**+**	**+**	**-**	**+**	**-**	**+**
H2052	**+**	**+**	**-**	**+**	**-**	**+**
MSTO 211 H	**+**	**+**	**-**	**+**	**-**	**+**
**prostate cancer** [169]						
PZ-HPV-7	**+**	**+**	**+**	**-**	**+**	**+**
LNCaP	**+**	**+**	**+**	**-**	**+**	**+**
DU145	**+**	**+**	**+**	**-**	**+**	**+**
PC3	**+**	**+**	**+**	**-**	**+**	**+**
**gastric cancer** [170]						
T	**-**	**+**	**-**	**+**	**+**	**+**
A	**-**	**+**	**-**	**+**	**+**	**+**
N	**-**	**+**	**-**	**+**	**+**	**+**

T: gastric tumor; A: adjacent non-neoplastic gastric tissue from patients with gastric cancer; N: non-neoplastic gastric tissue from individuals without gastric cancer

**Fig. 3:**
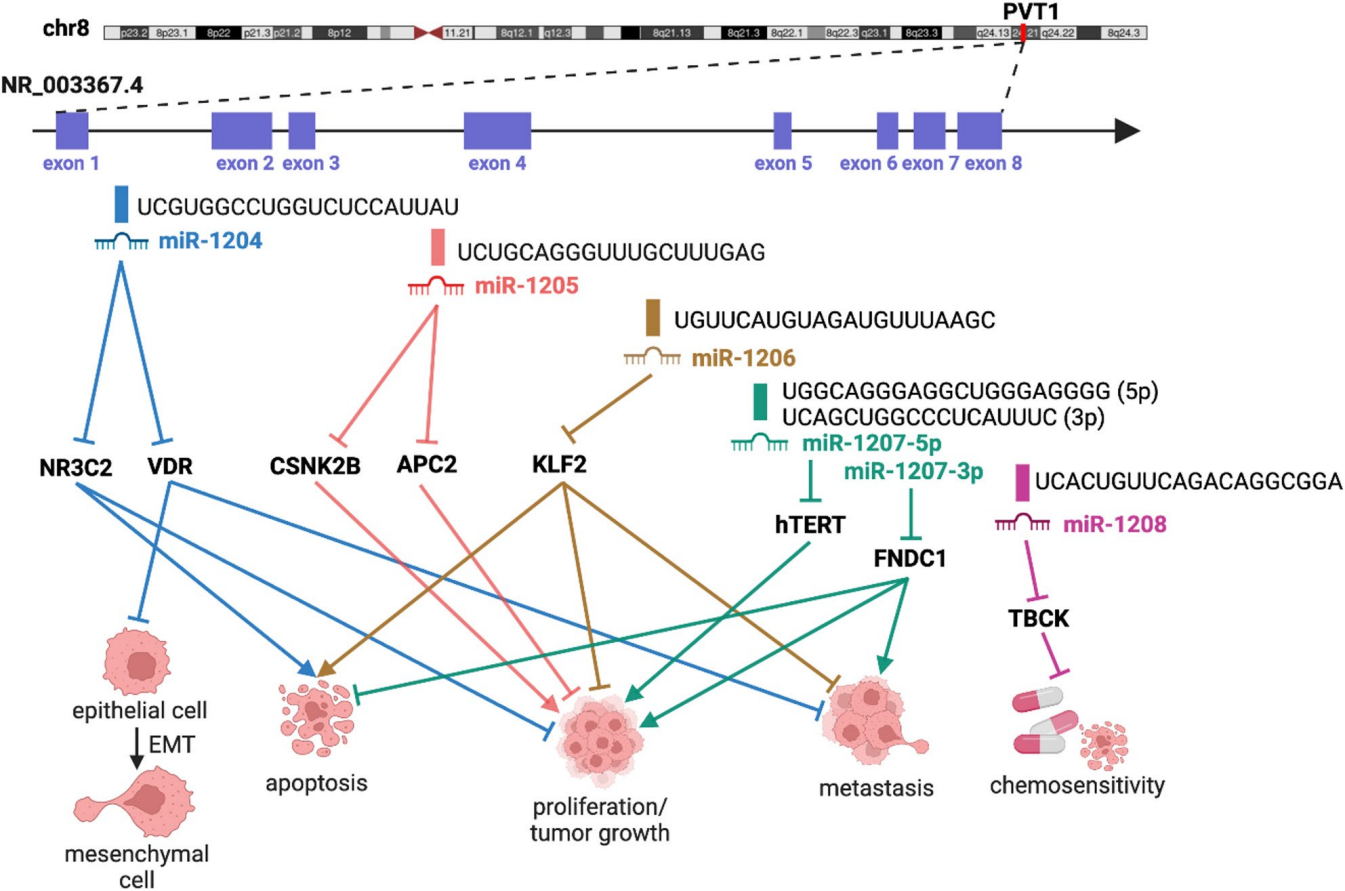
MiRNAs Encoded by the PVT1 Gene Locus: Implications in Cancer and Beyond. Diagram illustrating the PVT1 gene locus (the chromosome 8q24 region), PVT1 transcript NR_003367.4 and miRNAs encoded by the PVT1 gene locus. The encoding sites for miR-1204, miR-1205, miR-1206, miR-1207-3p, miR-1207-5p, and miR-1208 (indicated by colored bars) are depicted relative to exons and introns of PVT1 transcript NR_003367.4. The diagram displays the sequences of mature miRNAs and their target mRNAs, along with their regulatory effects on cancer cell phenotypes

These miRNAs have been reported to target genes involved in tumorigenesis, metastasis, and therapeutic resistance. Through the regulation of these target genes, miRNAs encoded by PVT1 can influence cancer cell behavior, affecting proliferation, invasion, and resistance to programmed cell death. For instance, miR-1204 induces a malignant phenotype in glioblastoma by targeting NR3C2 [171] and promotes epithelial-mesenchymal transition and metastasis in breast cancer by targeting VDR [172]. MiRNA-1205 enhances lung adenocarcinoma cell growth by targeting APC2 [173]. Elevated levels of miR-1206 in lung cancer accelerate cell proliferation, migration, and invasion by targeting KLF2 [174] (Fig. 3).

Similar to lncRNA PVT1, which is typically considered to play an oncogenic role, PVT1-encoded miRNAs can, under specific conditions, exert tumor-suppressive effects. While these miRNAs are known for their pro-cancer effects, they have also been documented to demonstrate anti-cancer properties. Interestingly, the anti-cancer actions of these miRNAs appear to outweigh those of lncRNA PVT1, depending on whether their target mRNA is pro- or anti-cancerous. For example, miRNA-1205 inhibits hepatocellular carcinoma cell proliferation through targeting CSNK2B [175]. In prostate cancer cells, miRNA-1207-3p suppresses proliferation and migration while promoting apoptosis by targeting FNDC1 [176]. Meanwhile, miR-1207-5p inhibits gastric tumor growth both in vitro and in vivo by targeting hTERT [177]. MiR-1208 enhances sensitivity to cisplatin in renal cancer cells by targeting TBCK [178] (Fig. 3).

### 8q24 rearrangements

PVT1 serves as a pivotal juncture for the 8q24 rearrangements, typically with accompanying high-copy number amplification of this region. Chimeras formed by gene rearrangements may be promoter-swapping fusions, two 3′UTR swapping events, or fusions that lead to gene truncation [31]. Through rearrangements, PVT1 triggers aberrant expression of downstream target genes, leading to the overactivation of oncogenes and the suppression of tumor suppressor genes, thereby promoting tumor development and progression.

The fusion gene PVT1-NSMCE2 has been identified in acute myelogenous leukemia, where it is encompassed within the segmental genome amplification of 8q24 [179]. PVT1-NBEA and PVT1-WWOX have been identified as highly expressed chimeric genes in multiple myeloma [180]. PVT1-NBEA chimeric transcripts result from the fusion of 5′-PVT1 exon 1 with NBEA exon 3–3′, while NBEA-PVT1 chimeric transcripts fuse 5′-NBEA exon 2 with PVT1 exon 3–3′. The PVT1-WWOX chimeric transcript forms by combining 5′-PVT1 exon 1 with WWOX exon 9 − 3′ [180].

Gene rearrangements involving the PVT1 locus also occur in solid tumors [181, 182]. Fusion genes within small cell lung cancers were detected using whole-transcriptome sequencing across 19 fresh tumors and 23 cell lines, in which PVT1 emerged as a recurrent 5’ partner gene within fusion transcripts, engaging with MYH7, CHD7, SLC7A7, CCNB1IP1, NOL4, CLVS1, and LY6H as 3’ partner genes [183]. The oncogenic PVT1/MYC fusion gene stands out as the sole recurrent PVT1 chimera found in solid tumors of diverse origins, including breast adenocarcinoma, colon adenocarcinoma, esophageal adenocarcinoma, and medulloblastoma [184]. Including PVT1 chimeras, chimeric genes could aid in identifying specific cancer subtypes and improving the treatment outcomes for patients affected by various types of tumors.

### Regulating protein stability and activity

**Fig. 4:**
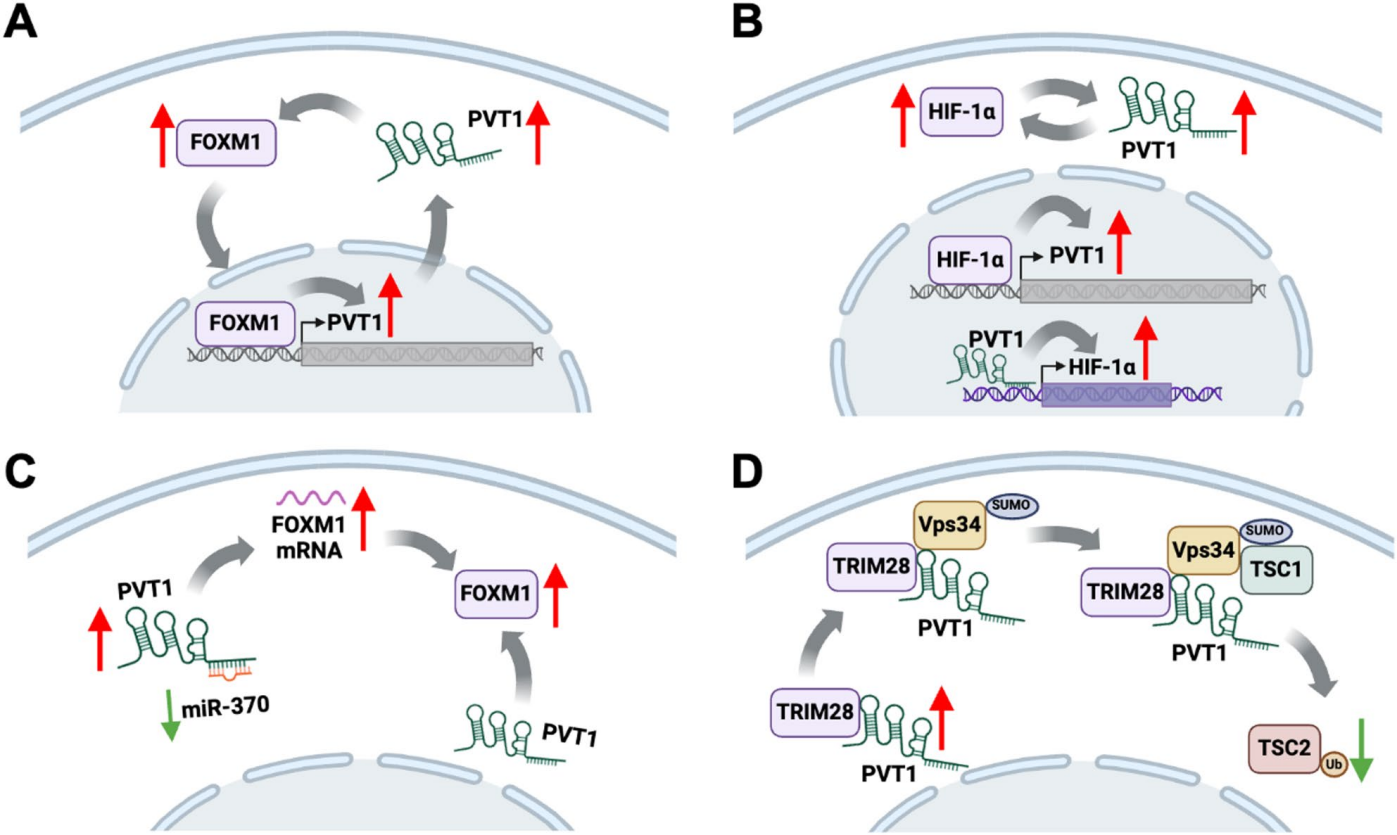
LncRNA PVT1 exerts function through regulating protein stability and activity. **A** PVT1 establishes positive feedback loops by binding to and stabilizing transcription factors, where the transcription factor, such as FOXM1 in gastric cancer, enhances the transcription of PVT1. **B** In pancreatic cancer, the PVT1-HIF-1α loop takes shape as PVT1 promotes HIF-1α transcription and stabilizes the protein, while concurrently, HIF-1α enhances the transcription and stabilization of PVT1 lncRNA. **C** PVT1 promotes gene expression through dual mechanisms: as a miRNA sponge stabilizing mRNA and binding to stabilize proteins. In ovarian cancer, it competitively binds miR-370 to stabilize FOXM1 mRNA and directly binds to stabilize FOXM1 protein. **D** The PVT-1/TRIM28 complex facilitates the SUMOylation and activation of Vps34, initiating the recruitment of TSC1 to Vps34 while dissociating TSC2 from the TSC1/TSC2 heterodimer. The released TSC2 undergoes ubiquitination, leading to its degradation

LncRNAs play a pivotal role in modulating protein stability and activity, which represents a significant mode of action. Their mechanism involves direct interaction with proteins, thereby influencing their stability and protecting them from degradation. For instance, PVT1 exemplifies this phenomenon through the formation of positive feedback loops with key transcription factors. In gastric cancer, PVT1 binds to and stabilizes transcription factors such as FOXM1 [185] (Fig. 4A); in bladder cancer, STAT5B [186]; and in clear cell renal cell carcinoma, HIF-2α [187], consequently amplifying PVT1 transcription. In pancreatic cancer, the PVT1-HIF-1α loop manifests wherein PVT1 not only boosts HIF-1α transcription and stabilizes the protein but also, reciprocally, HIF-1α augments the transcription and stabilization of PVT1 lncRNA [188] (Fig. 4B). To augment its regulatory influence, PVT1 facilitates gene expression through dual mechanisms: serving as a miRNA sponge to enhance mRNA stability and directly binding to stabilize proteins. In ovarian cancer, it competes for binding with miR-370, thereby stabilizing FOXM1 mRNA, while also directly binding to stabilize FOXM1 protein [115] (Fig. 4C). Similarly, in colorectal cancer, it competes with miR-128 to stabilize Lin28 mRNA and directly binds to stabilize Lin28 protein [76]. Moreover, in osteosarcoma, PVT1 competitively interacts with miR-183-5p to stabilize ERG mRNA and directly binds to stabilize ERG protein [93]. Additionally, in prostate cancer, it competes with miR-27b-3p to stabilize BLM mRNA and directly binds to stabilize BLM protein [67].

Apart from direct binding, PVT1 can also regulate protein stability by modulating the activity of intermediate proteins. In osteosarcoma, the PVT-1/TRIM28 complex promotes the SUMOylation and activation of Vps34, thereby initiating the recruitment of TSC1 to Vps34 and simultaneously dissociating TSC2 from the TSC1/TSC2 heterodimer. Subsequently, the liberated TSC2 undergoes ubiquitination, ultimately resulting in its degradation [189] (Fig. 4D).

In cancer cells, PVT1 regulates the stability of proteins, impacting the expression of oncogenes and tumor suppressor genes, thereby regulating key biological processes such as cell proliferation, metastasis, and treatment resistance, which is of significant importance in the regulation of cancer occurrence and development.

### Regulating transcription

**Fig. 5:**
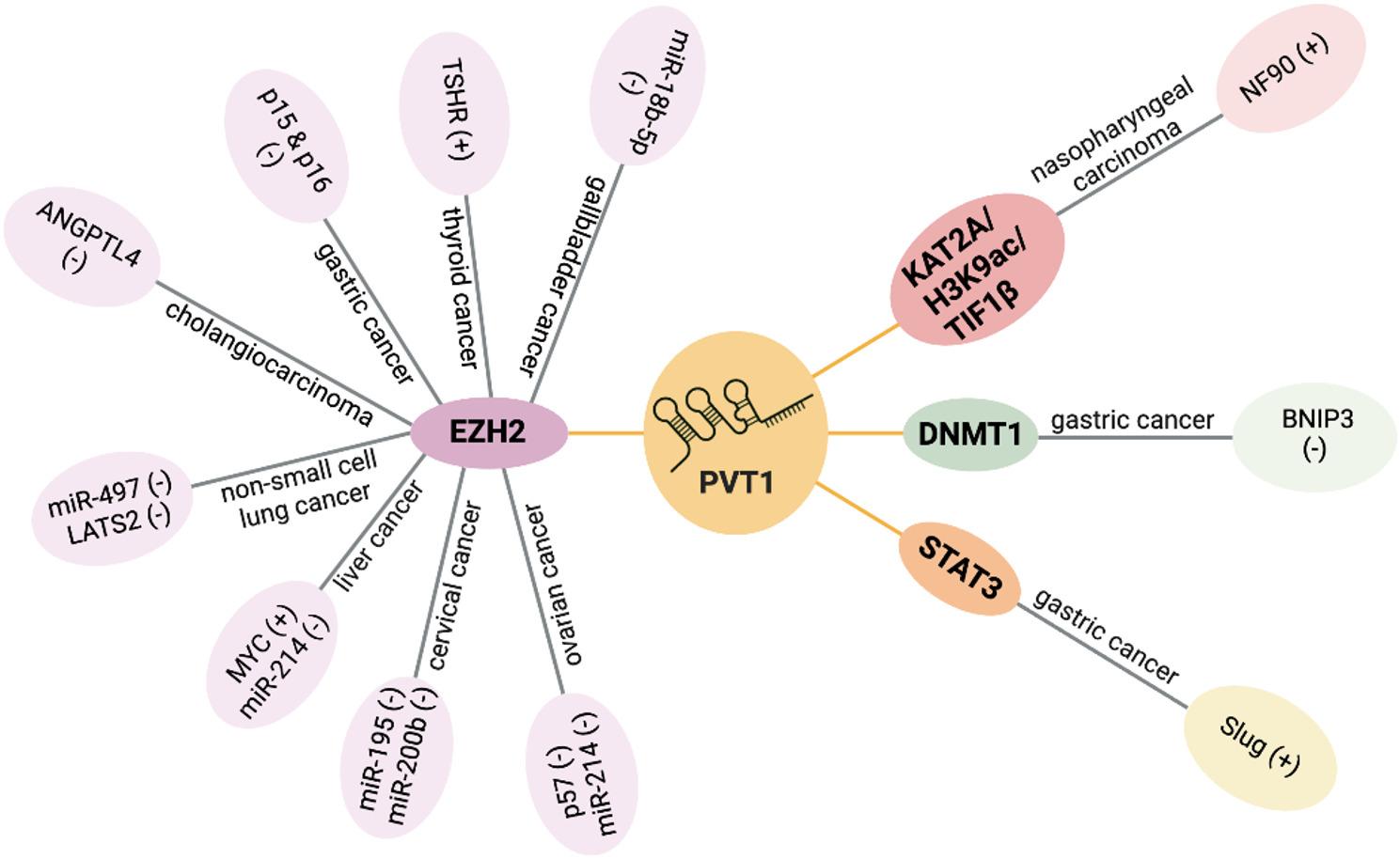
LncRNA PVT1 exerts function through regulating transcription. PVT1 interacts with EZH2 and plays a role in transcriptional regulation across various cancer types: in ovarian cancer, it downregulates the expression of p57 and miR-214; in cervical cancer, it suppresses the expression of miR-195 and miR-200b; in liver cancer, it inhibits miR-214 while promoting MYC expression; in non-small cell lung cancer, it decreases the expression of miR-497 and LATS2; in cholangiocarcinoma, it reduces ANGPTL4 expression; in gastric cancer, it suppresses p15 and p16 expression; in thyroid cancer, it upregulates TSHR expression, while in gallbladder cancer, it inhibits miR-18b-5p expression. In nasopharyngeal carcinoma, PVT1 binds to and facilitates the acetyltransferase activity of KAT2A, thereby enhancing the TIF1β/H3K9ac complex-mediated NF90 transcriptional activation. In gastric cancer, PVT1 downregulates BNIP3 transcription by increasing the methylation level of the BNIP3 promoter DNA through its interaction with the DNA methyltransferase DNMT1. In gastric cancer, PVT1 facilitated the recruitment of STAT3 to the Slug promoter, thereby transcriptionally enhancing Slug expression

An additional mechanism by which lncRNAs regulate gene expression is by controlling transcription (Fig. 5). For instance, PVT1 can interact with and modulate the function of epigenetic regulators, subsequently leading to either the activation or suppression of target gene expression. Enhancer of zeste homolog 2 (EZH2) is a significant protein enzyme that interacts with PVT1. It functions as the catalytic subunit of the Polycomb Repressive Complex 2 (PRC2), which modulates gene expression by facilitating the trimethylation of Lys-27 in histone 3 (H3K27me3) [190]. The interplay between PVT1 and EZH2 plays a crucial role in modulating the expression of various target genes across different cancer types. For instance, in ovarian cancer, it downregulates the expression of p57 [191] and miR-214 [192]. In cervical cancer, it suppresses miR-195 [193] and miR-200b [194] expression. In liver cancer, it inhibits miR-214 [195] while activating MYC [196] expression. In non-small cell lung cancer, it hampers miR-497 [197] and LATS2 [198] expression. In cholangiocarcinoma, it suppresses ANGPTL4 expression [40]. In gastric cancer, it diminishes p15 and p16 expression [41]. In thyroid cancer, it promotes TSHR expression [199], and in gallbladder cancer, it restrains miR-18b-5p expression [42]. Apart from its role in modulating transcription through interaction with EZH2, PVT1 activates NF90 transcription via the KAT2A/H3K9ac/TIF1β pathway in nasopharyngeal carcinoma [44]. In gastric cancer, PVT1 binds to the DNA methyltransferase DNMT1, thereby suppressing BNIP3 expression [200], or interacts with the transcription factor STAT3 to enhance Slug transcription [201].

## Functional elements of PVT1

The PVT1 gene locus encodes numerous isoforms, which are implicated in a wide range of physiological and pathological processes. These isoforms may have distinct roles depending on the biological context, yet the specific functional elements of PVT1 remain poorly characterized. Known PVT1 functional elements include exon 9, which is associated with prostate cancer and triple-negative breast cancer, and exon 2, which is linked to cSCC (Table 4).

Ogunwobi, O.O., et al. measured the expression levels of 12 exons of PVT1 and found that exon 9 was consistently and significantly overexpressed in aggressive prostate cancer cell lines derived from males of African ancestry [202]. Subsequent studies revealed that PVT1 exon 9 was upregulated in prostate cancer tissues, where it promoted cell proliferation and migration and contributed to the development of castration resistance by regulating the expression of proliferating cell nuclear antigen (PCNA) [203].

In addition, Ogunwobi, O.O., et al. reported that PVT1 exon 9 was upregulated in claudin-low triple-negative breast cancer and siRNA-mediated targeting of PVT1 exon 9 resulted in a marked decrease in migration and restored claudin 4 expression [204].

In cSCC, PVT1 exon 2 is reported to play a critical role in driving oncogenicity. PVT1 is upregulated in cSCC in situ and cSCC under the regulation of MYC oncogene. CRISPR-Cas9-mediated deletion of PVT1 exon 2 suppresses cSCC tumor growth both in vivo and in vitro [34].

**Table 4:** The functional elements of PVT1

Cancer	Functional elements	Expression	Function	Reference
prostate cancer	exon 9	increased in aggressive prostate cancer cell lines derived from males of African ancestry		[202]
prostate cancer	exon 9	increased in prostate cancer tissues	facilitate cell proliferation and migration while contributing to the development of castration resistance	[203]
breast cancer	exon 9	increased in caludin-low triple negative breast cancer	promote cell migration	[204]
cutaneous squamous cell carcinoma	exon 2	increased in cSCC in situ and cSCC	promote cell growth, colony formation and tumour sphere formation while suppress cellular senescence; promote tumor growth in vivo	[34]

Identifying and characterizing these elements is critical for understanding the biological significance of PVT1 and unlocking its clinical potential. Such insights could drive the development of targeted diagnostics and therapies, advancing the field of precision medicine.

## Clinical implications of PVT1

Elevated PVT1 expression is a robust biomarker for predicting tumor progression and patient outcomes across various cancers. Studies have demonstrated its potential to assess invasiveness in breast cancer [78], determine nodal metastasis in gastric cancer [205], evaluate histologic grade and nodal involvement in non-small-cell lung cancer [206], classify clinical stage and N status in pancreatic ductal adenocarcinoma [128], estimate the risk of recurrence in hepatocellular carcinoma [207], and predict poor prognosis in almost all types of cancer [208, 209]. Although the role of PVT1 in diagnosis and prognosis is still in the preclinical research stage, the diverse oncogenic isoforms produced by the PVT1 locus (such as lncRNA PVT1 subtypes, derived microRNAs like miR-1204, and circRNAs such as circPVT1) highlight its potential as a biomarker. These molecules, characterized by stability, tissue specificity, and detectability in body fluids, can overcome the limitations of traditional markers and support their potential for non-invasive tumor screening, treatment monitoring, and recurrence prediction, which are areas that require further investigation.

PVT1 has also emerged as a promising therapeutic target in cancer treatment. Its overexpression has been linked to resistance to chemotherapy, while its suppression increases the sensitivity of cancer cells to these treatments. In gastric cancer cell lines, for example, PVT1 was significantly increased in paclitaxel-resistant SGC7901 cells compared to untreated SGC7901 cells, suggesting that elevated PVT1 expression may serve as an indicator of paclitaxel resistance [205]. Similar results were observed in cisplatin-resistant SGC7901/DDP and BGC823/DDP gastric cancer cell lines, in which PVT1 deletion effectively reversed cisplatin resistance [210]. In human pancreatic cancer, PVT1 was shown to regulate negatively gemcitabine sensitivity in ASPC-1 cells [209]. Furthermore, in the malignant pleural mesothelioma cell line MSTO-211 H, increased sensitivity to cisplatin was observed after treatment with siMYC and siPVT1, highlighting the role of PVT1 depletion in enhancing responses to chemotherapy [30].

Similarly, PVT1-derived products such as circPVT1 and some derived miRNAs, have demonstrated significant clinical relevance. For example, circPVT1 has been shown to be upregulated in both osteosarcoma tissues and serum of affected patients, which correlates with poor prognosis [211]. In addition, circPVT1 expression has been significantly elevated in patients with chemoresistant osteosarcoma compared to chemosensitive ones [149]. Likewise, in gastric cancer, serum exosomal levels of circPVT1 have been found to be higher in cisplatin-resistant patients [212]. Meanwhile, down-regulation of miR-1207-5p has been closely associated with malignant progression of laryngeal squamous cell carcinoma (LSCC) [213]. Furthermore, lower plasma levels of miR-1207-5p have been associated with shorter overall survival in patients with colorectal cancer [214]. miR-1206 has been associated with a poor prognosis in ovarian cancer [215]. Overexpression of miR-1204 has been identified as an independent prognostic factor associated with poor outcomes in breast cancer [216]. In contrast, elevated expression of miR-1205 is linked to a favorable prognosis in patients with LSCC [217].

## Discussion

The PVT1 gene exhibits remarkable complexity, particularly due to its large size and its ability to produce numerous isoforms, which has implications for diverse cellular processes and cancer types. PVT1 is involved in an unusual variety of regulatory pathways, primarily through interactions with other RNAs, proteins, and chromatin structures. This gene size contributes to its ability to encode dozens of alternatively spliced isoforms, each potentially having distinct functions or regulatory roles. This alternative splicing allows PVT1 to interact with numerous molecular targets, further diversifying its influence on cellular processes. In this work, we identified 74 distinct miRNAs sponged by linear PVT1 and 26 miRNAs sponged by circPVT1, as detailed in Fig. 2, Supplementary Tables 1, and Table 2.

In cancer, PVT1’s function varies widely depending on the tissue and cellular context, contributing to its characterization as an oncogenic driver. In some cancers, such as breast, lung, and colorectal, PVT1 has been observed to promote cell proliferation and inhibit apoptosis by modulating the expression of key oncogenes and tumor suppressors. One prominent mechanism involves interactions with the MYC oncogene; PVT1 enhances MYC stability and activity, thereby promoting tumor growth. In other cancers, such as ovarian and hepatocellular carcinomas, PVT1 can act through different pathways, including influencing miRNAs and sponging tumor-suppressive miRNAs to derepress oncogenic pathways [87, 97, 114, 138, 141]. Furthermore, PVT1 exhibits functional versatility through its involvement in epigenetic regulation, altering chromatin states to favor oncogenic gene expression. It recruits chromatin-modifying complexes, such as PRC2, which helps maintain the cancer cell phenotype [218]. These multifaceted roles highlight PVT1 as both a potential therapeutic target and a biomarker for various cancers. Its ability to engage in multiple mechanisms across cancers underscores the gene’s adaptability and complex involvement in oncogenesis, with ongoing research revealing ever more layers of functional diversity and regulatory potential.

In general, the mechanism of action of lncRNAs is often intricately linked to their subcellular localization, as this determines their ability to interact with specific molecular partners and influence cellular processes. PVT1 exhibits dynamic subcellular localization patterns that contribute to its diverse functions in cancer. PVT1 can be found in two main places in the cell (the nucleus and the cytoplasm), and in each location, it has different roles. When PVT1 is predominantly localized in the nucleus it participates in chromatin remodeling and gene expression regulation. For example, in breast cancer cells, PVT1 has been found to interact with PRC2 in the nucleus, influencing gene silencing and promoting tumorigenesis [218]. In the cytoplasm, PVT1 often acts as a “sponge” that soaks up miRNAs - small molecules that usually stop the production of certain proteins. For instance, in lung cancer cell lines, cytoplasmic PVT1 sequesters miR-128 and miR-497, preventing them from inhibiting the expression of oncogenes like VEGFC and BCL-2, which enhances cell survival and proliferation [80, 165]. Notably, the different transcripts of PVT1 may localize distinctly. CircPVT1 is predominantly localized in the cytoplasm, while most linear transcripts of PVT1 are primarily found in the nucleus. This suggests that different PVT1 transcripts may perform functions specific to their respective subcellular locations [37]. These findings highlight the importance of understanding PVT1’s subcellular localization as a key factor in determining its mechanistic role in different cancer types, as its position within the cell dictates its ability to interact with specific molecular pathways and modulate oncogenic processes.

The multiple exons in PVT1 give rise to many alternative splicing events, resulting in different transcript variants that may exert distinct cellular effects. These variants contribute to PVT1’s flexibility in influencing various signaling pathways, and their differential expression is often linked to specific types of cancers. One of the notable features of PVT1 is that it encodes several miRNAs within its sequence, such as miR-1204, miR-1205, miR-1206, miR-1207, and miR-1208 (see Fig. 3). These miRNAs often target tumor-suppressor genes or other regulatory genes involved in cell cycle control, apoptosis, and metastasis. The miRNAs derived from PVT1 can act independently or synergistically with PVT1 lncRNA to drive oncogenic processes, adding a layer of complexity to its regulatory potential. PVT1 functions as a competing endogenous RNA (ceRNA) or ‘miRNA sponge’: by “sponging” or binding to tumor-suppressive miRNAs, reducing their availability to bind their target mRNAs. This sponging function essentially protects oncogenic transcripts from being degraded, supporting tumor growth. For example, PVT1 can sequester miRNAs like miR-200 and miR-186, which otherwise would suppress cancer-associated genes [94–96, 105]. The PVT1 locus contains promoter and enhancer regions that interact with other oncogenic loci, most notably MYC, which is adjacent to PVT1 on chromosome 8q24. This region is frequently amplified in cancers, and the enhancers at the PVT1 locus can drive MYC expression. Through enhancer-mediated looping, PVT1 and MYC form a regulatory axis, which amplifies oncogenic signaling pathways when PVT1 is upregulated. PVT1 binds to various proteins, including transcription factors, chromatin modifiers, and RNA-binding proteins. For instance, PVT1 can recruit EZH2, a component of the PRC2 complex, to methylate histones and repress tumor-suppressor genes [219]. This epigenetic modification supports a pro-cancerous environment. PVT1 also interacts with MYC, helping to stabilize the MYC protein by preventing its ubiquitination and degradation, a mechanism that underscores its role in MYC-driven cancers. Although it is a non-coding RNA, PVT1 has specific secondary and tertiary structures that are crucial for its interactions with other RNAs, DNA, and proteins. These structural elements help facilitate its role as a scaffold in various molecular complexes, which can lead to chromatin remodeling and alterations in gene expression.

PVT1 binds to a remarkable number of specific miRNAs to modulate the expression of target genes, driving cancer progression by affecting cellular proliferation, invasion, migration, and resistance to apoptosis. We have collected information about 100 specific RNAs that are listed in Supplementary Tables 1 and Table 2. The Table also shows that PVT1 influences cancer development across diverse tissues, making it a potential target for therapeutic interventions across multiple cancer types. Its regulatory effects on miRNAs position could be a critical factor in tumor progression and drug resistance. We summarize below these data on some critical cancer categories. In renal and urological cancers, PVT1’s interaction with miR-15b in clear cell renal cell carcinoma enhances stem-like properties and promotes angiogenesis, while in bladder cancer, its modulation of miR-31 and miR-128 fosters cell proliferation, migration, and angiogenesis, driving tumor progression [61, 71, 75]. In clear cell renal cell carcinoma, PVT1 interacts with miR-15b to upregulate the VEGFR2 gene (KDR), thereby enhancing stemness and angiogenesis, promoting tumorigenesis in vivof, in prostate cancer, PVT1 sponges miR-15a-5p to increase KIF23 levels, which stimulates cell proliferation, invasion, and reduces apoptosis, resulting in increased tumor growth. PVT1 also impacts prostate cancer by sponging miR-15a-5p and miR-27b-3p, enhancing proliferation and invasiveness [62, 67]. In gastrointestinal cancers such as colorectal, gastric, pancreatic, and esophageal cancers, PVT1 plays a critical role. By sponging miR-16-5p and miR-106b-5p, it promotes tumor growth and metastasis in colorectal cancer, while in gastric cancer, its interactions with miR-186 and miR-16 increase cell proliferation and invasion [63, 64, 73, 95]. Lung cancer, particularly non-small cell lung cancer, also shows a strong connection to PVT1, which modulates miR-29c, miR-140-3p, and miR-181a-5p to enhance angiogenesis, autophagy, and resistance to chemotherapy, crucial for cancer cell survival and metastasis [68, 82, 92]. In hepatocellular and liver cancers, PVT1 targets miR-214-3p, miR-1258 and miR-3619-5p, promoting proliferation, migration, and apoptosis resistance, supporting liver tumor growth and metastasis [108, 138, 141]. Brain cancers, like glioma, involve PVT1 interactions with miR-128-3p, miR-186, and miR-1207-3p, which lead to increased proliferation, migration, and angiogenesis, contributing to aggressive tumor growth and chemotherapy resistance [79, 96, 137]. Additionally, PVT1 influences breast, ovarian, thyroid, and endometrial cancers by regulating miRNAs such as miR-128-3p, miR-543, and miR-612, thereby promoting proliferation, metastasis, and therapy resistance. In sum, PVT1’s broad regulatory effects on miRNAs highlight its role in cancer progression across diverse tissues, positioning it as a promising target for therapeutic interventions across multiple cancer types.

Although PVT1 is frequently characterized as a miRNA sponge through ceRNA mechanisms, this paradigm faces substantial scrutiny. The efficacy of the ceRNA mechanism is critically dependent on the number and distribution of miRNA response elements (MREs), as well as the relative abundance of ceRNAs and their target miRNAs [220]. Quantitative studies demonstrate that effective miRNA sequestration requires ceRNA expression exceeding target miRNA levels—a condition rarely satisfied physiologically [221]. Furthermore, transcriptome-wide analyses further indicate that most lncRNAs lack sufficient MREs density to broadly perturb endogenous miRNA activity [222]. Elevated PVT1 expression resulting from either 8q24 amplification-driven PVT1/MYC co-overexpression or alternative regulatory mechanisms constitutes a common occurrence in cancers. Nevertheless, rigorous evaluation of PVT1’s purported miRNA sponge function should involve: (1) quantitative comparisons of endogenous PVT1 and target miRNA stoichiometry, and (2) validation extending beyond artificial overexpression systems.

Several genes share structural and functional similarities with PVT1, especially those involved in complex regulatory roles and multi-isoform expression in cancer biology. For instance, the MALAT1 (Metastasis-Associated Lung Adenocarcinoma Transcript 1) gene, is a well-known lncRNA, that exhibits a comparable complexity due to its large size and its ability to produce various isoforms [223]. MALAT1 has been implicated in alternative splicing and gene regulation through interactions with splicing factors and RNA-binding proteins, which can contribute to oncogenesis and metastasis in multiple cancers, including lung, liver, and breast cancers. Like PVT1, MALAT1 can serve as a molecular sponge, modulating miRNAs to influence cellular proliferation, migration, and invasion pathways relevant to tumor development. Another gene, HOTAIR (HOX Transcript Antisense RNA), also demonstrates a complex regulatory role in gene expression and epigenetic modification [224]. HOTAIR interacts with polycomb repressive complexes to alter chromatin states, repressing specific gene expression in a manner that promotes tumor progression and metastasis in cancers such as breast and liver. Its large size and ability to bind to multiple proteins, much like PVT1, enable it to modulate various cellular pathways, including epithelial-to-mesenchymal transition (EMT) and apoptosis resistance, making it a significant player in cancer metastasis. Additionally, NEAT1 (Nuclear Enriched Abundant Transcript 1), which produces different isoforms that contribute to the structure and function of paraspeckles, plays a role in stress response, RNA stability, and gene regulation [225]. Like PVT1, NEAT1 interacts with other RNAs and proteins, influencing processes like transcription regulation and cellular stress responses, and its dysregulation has been linked to cancer and inflammatory diseases. These genes, like PVT1, demonstrate structural diversity and functional versatility, positioning them as potential therapeutic targets in oncology due to their roles in cellular transformation and disease progression.

Certain pollutants associated with cancer development have been shown to change the expression of PVT1 or its related products. For example, exposure to fine particulate matter (PM2.5), a type of air pollutant with particles less than 2.5 micrometers in diameter, has been shown to elevate PVT1 levels. PVT1 acts as a molecular sponge for miR-199a, binding to it and suppressing its activity. Since miR-199a normally inhibits caveolin-1 signaling, its suppression by PVT1 leads to an increase in caveolin-1 activity. This, in turn, activates oncogenic pathways such as PI3K/AKT/mTOR and MAPK/ERK signaling, which drive cancer progression and promote cell survival. Also, PVT1 expression was observed to decrease following cadmium-induced oxidative damage at micromolar concentrations. This affects mRNA and miRNA processing and subsequently influences oxidative damage [226]. More speculatively, but pollutants such as polycyclic aromatic hydrocarbons (PAHs), heavy metals and endocrine-disrupting chemicals are known to induce oxidative stress, inflammation and DNA damage, conditions in which increased PVT1 expression has often been observed. This may suggest a possible role of PVT1 in the procancerogenic effects of these pollutants, although experimental confirmation is needed.

Given the critical role of PVT1 lncRNA in cancer pathogenesis, numerous studies have highlighted its potential as a therapeutic target in various malignancies. These include non-small cell lung cancer, hepatocellular carcinoma, gastric cancer, breast cancer and glioma, among others [227]. Targeting the functional elements of PVT1 offers several promising therapeutic approaches in cancer treatment. By blocking the miRNA-binding sites, it may be possible to free up tumor-suppressive miRNAs, which are often sequestered by PVT1, to inhibit oncogenic pathways. Small molecules or RNA-based therapeutics, such as antagomirs (antisense oligonucleotides against miRNAs) or miRNA mimics, are currently in development to modulate miRNA activity. For instance, antagomirs targeting oncogenic miRNAs like miR-17 have been shown to reduce tumor proliferation in preclinical models [228], which could be a relevant approach if applied to miRNAs associated with PVT1. This strategy may open doors to restoring miRNA function specifically in cancers where PVT1 activity leads to miRNA dysregulation. Another therapeutic avenue is directly targeting the proteins that interact with PVT1, such as MYC and EZH2. PVT1 has been shown to regulate MYC expression, which is crucial in numerous cancers, and EZH2, a histone methyltransferase, modulates gene expression patterns that are crucial for cancer progression. Small molecule inhibitors for MYC, though challenging due to MYC’s structure, have seen promising developments. For example, BET inhibitors like JQ1 disrupt MYC transcription indirectly by inhibiting the BET bromodomain proteins, which assist in MYC transcriptional activity [229]. Meanwhile, EZH2 inhibitors like Tazemetostat have shown efficacy in cancers with EZH2 mutations, and researchers are exploring whether these inhibitors could be useful in cancers where PVT1 modulates EZH2 [230]. Enhancer interactions also represent an appealing therapeutic target, given PVT1’s role in amplifying MYC expression via enhancer elements. Genome-editing tools such as CRISPR-Cas9 can be employed to selectively disrupt these enhancer regions. In certain leukemias, CRISPR has been used to edit enhancer regions directly, silencing oncogene expression, which could be similarly applied to PVT1-associated enhancers. CRISPR-interference techniques, where dCas9 fused with a transcriptional repressor can be used to downregulate specific enhancers [231], highlights the potential efficacy and safety of this approach for targeting MYC overexpression in cancer. In summary, by focusing on the specific roles of PVT1, whether through miRNA sponging, protein interactions, or enhancer regions, it is likely that novel therapies can be developed to disrupt its multifaceted contributions to cancer progression. These strategies show potential in creating targeted therapies that can offer greater specificity and reduce the side effects often seen in traditional cancer treatments.

## Supplementary Information

Supplementary Material 1

## Data Availability

No datasets were generated or analysed during the current study.
