# Supplementary material for: LncRNA PVT1 in human cancers: genomic complexity, isoforms, functional elements, mechanism of action, subcellular localization and possible role as a therapeutic target
Source: Mol Cancer. 2026 Jan 21;25:51. doi: 10.1186/s12943-026-02576-y (PMC12952124; doi:10.1186/s12943-026-02576-y)
Supplement: Supplementary file 1 — Supplementary Material 1 [file 12943_2026_2576_MOESM1_ESM.docx]

**Supplementary Table 1.** Linear PVT1 acts as miRNA sponges

| **Cancer** | **Interacting miRNA** | **Target gene** | **Function** | **References** |
| --- | --- | --- | --- | --- |
| clear cell renal cell carcinoma | miR-15b | KDR | induce stemness;  promote tumorigenesis and angiogenesis *in vivo* | ^61^ |
| prostate cancer | miR-15a-5p | KIF23 | promote proliferation, migration and invasion;  inhibit apoptosis;  promote tumor growth *in vivo* | ^62^ |
| colorectal cancer | miR-16-5p | VEGFA | promote proliferation, invasion, and migration;  promote tumor growth *in vivo* | ^63^ |
| gastric cancer | miR-16 | CCND1 | promote proliferation and invasion | ^64^ |
| uveal melanoma | miR-17-3p | MDM2 | promote proliferation, migration, and invasion;  activate p53 | ^65^ |
| colorectal cancer | miR-24-3p | NRP1 | promote proliferation and metastasis | ^66^ |
| prostate cancer | miR‑27b‑3p | BLM | promote proliferation, apoptotic resistance, invasion and migration;  promote tumor growth *in vivo* | ^67^ |
| non-small cell lung cancer | miR-29c | VEGF | promote angiogenesis | ^68^ |
| acute myeloid leukaemia | miR-29 family | WAVE1 | promote growth, migration and invasion;  inhibit apoptosis | ^69^ |
| colon cancer | miR-30d-5p | RUNX2 | promoted tumor growth and invasion | ^70^ |
| bladder cancer | miR-31 | CDK1 | promote growth, migration, and invasion;  promote tumor growth *in vivo* | ^71^ |
| diffuse large B-cell lymphoma | miR-34b-5p | Foxp1 | disinhibit Foxp1/β-catenin signaling;  promote cell proliferation;  inhibit apoptosis;  promote tumor growth *in vivo* | ^72^ |
| colorectal cancer | miR-106b-5p | FJX1 | promote proliferation migration and invasion;  promote tumor growth *in vivo* | ^73^ |
| lung cancer | miR-126 | SLC7A5 | promote proliferation | ^74^ |
| bladder cancer | miR-128 | VEGFC | promote proliferation and migration;  promote tumor growth *in vivo* | ^75^ |
| colorectal cancer | miR-128 | Lin28 | promote proliferation, stem-like properties, and metastasis;  promote tumor growth and metastasis *in vivo* | ^76^ |
| esophageal cancer | miR-128 | ZEB1 | promote proliferation, migration and invasion | ^77^ |
| breast cancer | miR-128-3p | FOXQ1 | promote proliferation, migration and invasion；  promote tumor growth and metastasis *in vivo* | ^78^ |
| glioma | miR-128-3p | GREM1 | promote proliferation, invasion, migration and cell cycle progression;  inhibit apoptosis;  promote tumor growth *in vivo* | ^79^ |
| lung cancer | miR-128 | VEGFC | promote proliferation and metastasis *in vitro* and *in vivo* | ^80^ |
| endometrial cancer | miR-136 | Sox2 | promote proliferation, migration, invasion, sphere formation and stemness;  inhibit apoptosis;  contribute to carboplatin resistance;  positively regulate UPF1 expression | ^81^ |
| lung cancer | miR-140-3p | ATG5 | promote autophagy;  contribute to cisplatin resistance *in vitro* and *in vivo* | ^82^ |
| cervical cancer | miR-140-5p | Smad3 | promote proliferation, migration and invasion | ^83^ |
| gallbladder cancer | miR-143 | HK2 | promote cell proliferation, migration, and invasion;  promote glucose metabolism;  promote tumor growth *in vivo* | ^84^ |
| pancreatic cancer | miR-143 | HIF-1α | promote autophagy;  contribute to gemcitabine resistance | ^85^ |
| breast cancer | miR-148a-3p | ROCK1 | promote migration and invasion | ^86^ |
| ovarian cancer | miR-148a-3p | AGO1 | promote proliferation and metastasis;  promote tumour growth *in vivo*;  activate TGF-β pathway;  activate ERK1/2, smad2 and smad4 | ^87^ |
| oral squamous cell carcinoma | miR‑150‑5p | GLUT‑1 | promote proliferation, invasion and migration;  inhibit apoptosis;  promote tumor growth and expression of invasion and migration-associated genes *in vivo* | ^88^ |
| gastric cancer | miR-152 | CD151 and FGF2 | - | ^89^ |
| colorectal cancer | miR‐152‐3p | E2F3 | promote proliferation, invasion and migration;  promote tumor growth *in vivo*;  promote the transcriptional activation of MAPK8 | ^90^ |
| esophageal carcinoma | miR181a-5p | GLS | contribute to cisplatin resistance | ^91^ |
| non-small cell lung cancer | miR181a-5p | SP1 | promote cell growth;  promote tumor growth *in vivo* | ^92^ |
| osteosarcoma | miR-183-5p | ERG | promotes proliferation and migration;  promote tumor growth and metastasis *in vivo* | ^93^ |
| cholangiocarcinoma | miR-186 | SEMA4D | promote proliferation, migration and invasion;  promote tumor growth *in vivo* | ^94^ |
| gastric cancer | miR-186 | HIF-1α | promote cell proliferation and invasion | ^95^ |
| glioma | miR-186 | Atg7 and Beclin1 | induce protective autophagy;  promote glioma vascular endothelial cell proliferation, migration, and angiogenesis | ^96^ |
| hepatocellular carcinoma | miR-186-5p | YAP1 | promote proliferation, invasion, and migration;  inhibite apoptosis | ^97^ |
| prostate cancer | miR-186-5p | Twist1 | promote invasion and metastasis | ^98^ |
| glioma | miR-190a-5p and miR-488-3p | MEF2C | promote proliferation, migration and invasion;  inhibit apoptosis;  promote tumor growth *in vivo*;  promote JAGGED1 expression by increasing the promoter activity of JAGGED1 | ^99^ |
| bladder cancer | miR-194-5p | BCLAF1 | promote proliferation, migration;  inhibit apoptosis;  promote tumor growth *in vivo* | ^100^ |
| oral squamous cell carcinoma | miR-194-5p | HIF1a | promote proliferation and cisplatin resistance | ^101^ |
| endometrial carcinoma | miR-195-5p | FGFR1 and FGF2 | activate PI3K/AKT and MAPK/Erk pathways;  promote cell proliferation, migration, and invasion;  inhibit apoptosis;  promote tumor growth *in vivo* | ^102^ |
| lung cancer | miR-199a | caveolin1 | increase the expression of IL6 and TNFα in PM2.5-exposed cells;  promote PM2.5-exposed cell EMT and migration | ^103^ |
| non‑small cell lung cancer | miR‑199a‑5p | HIF‑1α | promote proliferation | ^104^ |
| non‑small cell lung cancer | miR-200a and miR-200b | MMP9 | promote invasion | ^105^ |
| esophageal squamous cell carcinoma | miR-203 | LASP1 | promote proliferation and migration *in vitro*;  promote tumor growth *in vivo* | ^106^ |
| colorectal cancer | miR-214-3p | IRS1 | activate the PI3K/Akt signaling pathway;  promote proliferation and invasion;  inhibit apoptosis | ^107^ |
| liver cancer | miR-214-3p | GPX4 | promote cell growth;  inhibit ferroptosis | ^108^ |
| colorectal cancer | miR-216a-5p | YBX1 | tumor growth, metastasis, EMT | ^109^ |
| non-small cell lung cancer | miR-216b | Beclin-1 | promote autophagy;  inhibit apoptosis;  promote tumor growth *in vivo* | ^110^ |
| clear cell renal cell carcinoma | miR-328-3p | FAM193B | promote proliferation;  activate PI3K/AKT and MAPK/ERK signaling pathways | ^111^ |
| non-small-cell lung cancer | miR-361-3p | SOX9 | activate Wnt/β-catenin signaling pathway;  promote proliferation, migration, invasion;  inhibit apoptosis | ^112^ |
| glioma | miR-365 | ELF4 | induce stemness and temozolomide resistance | ^113^ |
| hepatocellular carcinoma | miR-365 | ATG3 | facilitate cell autophagy | ^114^ |
| ovarian cancer | miR-370 | FOXM1 | promote proliferation, migration and invasion | ^115^ |
| head and neck squamous cell carcinoma | miR-375 | YAP1 | promote proliferation, stemness and metastasis;  promote tumor growth *in vivo*;  inhibit anti-tumor immunity;  contribute to resistance to PD1 blockade therapy;  facilitate the cellular DNA damage response | ^46^ |
| lung adenocarcinoma | miR-378c | SLC2A1 | promote proliferation, migration, and invasion | ^116^ |
| pancreatic cancer | miR-409 | SHH | activate the SHH/GLI/MGMT signaling pathway;  promote autophagy;  inhibit apoptosis;  promote tumor growth *in vivo* | ^117^ |
| thyroid cancer | miR-423-5p | PAK3 | promote proliferation and invasion;  inhibit apoptosis | ^118^ |
| colorectal cancer | miR-455 | RUNX2, RAF-1 | proliferation, migration and invasion | ^119^ |
| cervical cancer | miR-486-3p | ECM1 | proliferation | ^120^ |
| acute lymphoblastic leukemia | miR-486-5p | MAML3 | inhibit apoptosis;  promote tumor growth *in vivo* | ^121^ |
| colon cancer | miR-486-5p | CDK4 | contribute to 5-FU resistance | ^122^ |
| retinoblastoma | miR-488-3p | Notch2 | promote proliferation, migration, invasion, and cell cycle progression;  inhibit apoptosis;  promote tumor growth *in vivo* | ^123^ |
| osteosarcoma | miR-497 | HK2 | promote glucose uptake, lactate production;  promote cell proliferation, cell cycle progression, and invasion | ^124^ |
| cervical cancer | miR-503 | ARL2 | promote cell viability, migration and invasion;  inhibit apoptosis;  promote tumor growth *in vivo* | ^125^ |
| nasopharyngeal carcinoma | miR-515-5p | PIK3CA | promote proliferation;  inhibit apoptosis;  contribute to radioresistance | ^126^ |
| prostate cancer | miR-515-5p | HMGB3 | promote proliferation, migration, and invasion | ^127^ |
| pancreatic ductal adenocarcinoma | miR-519d-3p | HIF-1A | promote proliferation, invasion and glycolysis;  promote tumor growth *in vivo* | ^128^ |
| non-small cell lung cancer | miRNA-526b | EZH2 | promote cell proliferation and migration | ^129^ |
| breast cancer | miR-543 | TRPS1 | promote growth, motility;  inhibit apoptosis | ^130^ |
| ovarian cancer | miR-543 | SERPINI1 | promote proliferation, migration and invasion;  inhibit apoptosis | ^131^ |
| non-small cell lung cancer | miR-551b | FGFR1 | promote viability, proliferation, migration and invasion | ^132^ |
| advanced endometrial cancer | miR-612 | CENP-H | promote cell proliferation, migration, invasion;  inhibit cell apoptosis;  promote tumor growth *in vivo*;  activate Akt/mTOR signaling pathway | ^133^ |
| pancreatic cancer | miR-619-5p | Pygo2 and ATG14 | activate Wnt/β-catenin signaling pathway;  promote autophagic activity;  contribute to gemcitabine resistance *in vitro* and *in vivo* | ^134^ |
| non-small cell lung cancer | miR-760 | IL-6 | promote migration and invasion | ^135^ |
| colorectal cancer | miR‑761 | MAPK1 | promote proliferation;  inhibit apoptosis | ^136^ |
| glioma | miR-1207-3p | HNF1B | promote proliferation, migration, invasion and angiogenesis;  promote tumor growth *in vivo* | ^137^ |
| hepatocellular carcinoma | miR‐1258 | DUSP13 | regulate lipid metabolism in the microvascular invasion process | ^138^ |
| laryngeal squamous cell carcinoma | miR-1301-3p | MBNL1 | promote proliferation;  inhibit apoptosis;  increase the susceptibility to natural killer cells | ^139^ |
| gastric cancer | miR-3619-5p | TBL1XR1 | contribute to cisplatin resistance;  promote viability, apoptosis, migration, and invasion;  promote tumor growth *in vivo*; | ^140^ |
| hepatocellular carcinoma | miR-3619-5p | MKL1 | promote migration | ^141^ |
